# A new view of axon growth and guidance grounded in the stochastic dynamics of actin networks

**DOI:** 10.1098/rsob.220359

**Published:** 2023-06-07

**Authors:** Rameen Forghani, Aravind Chandrasekaran, Garegin Papoian, Edward Giniger

**Affiliations:** ^1^ National Institute of Neurological Disorders and Stroke, National Institutes of Health, Bethesda, MD 20892, USA; ^2^ Department of Chemistry and Biochemistry, University of Maryland, College Park, MD 20742-3281, USA; ^3^ Institute for Physical Science and Technology, University of Maryland, College Park, MD 20742-3281, USA

**Keywords:** axon guidance, actin dynamics, stochastic process, growth cone, live imaging, computational simulation

## Abstract

The mechanism of axon growth and guidance is a core, unsolved problem in neuroscience and cell biology. For nearly three decades, our view of this process has largely been based on deterministic models of motility derived from studies of neurons cultured *in vitro* on rigid substrates. Here, we suggest a fundamentally different, inherently probabilistic model of axon growth, one that is grounded in the stochastic dynamics of actin networks. This perspective is motivated and supported by a synthesis of results from live imaging of a specific axon growing in its native tissue *in vivo*, together with single-molecule computational simulations of actin dynamics. In particular, we show how axon growth arises from a small spatial bias in the intrinsic fluctuations of the axonal actin cytoskeleton, one that produces net translocation of the axonal actin network by differentially modulating local probabilities of network expansion versus compaction. We discuss the relationship between this model and current views of axon growth and guidance mechanism and demonstrate how it offers explanations for various longstanding puzzles in this field. We further point out the implications of the probabilistic nature of actin dynamics for many other processes of cell morphology and motility.

## Graphical abstract

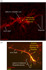

## 1. Introduction

At the single-cell level, biology is statistical. Macroscopic features of organisms, such as their morphology and physiology, tend to be remarkably consistent and predictable, but the cellular and subcellular machines that make those features show tremendous fluctuation and variability in their moment-to-moment activity [1–5]. One of the great challenges in contemporary cell biology is to understand the mechanisms by which noisy biochemistry and biophysics give rise to reliable biological outcomes.

In this Commentary, we will consider the implications of this challenge for the mechanism of axon growth and guidance in the developing nervous system. The function of a nervous system depends on its pattern of wiring, where the ‘wire’ that carries information from one neuron to those downstream in its neural circuit is called an axon. However, those wires do not start out having their complex final shapes, they have to grow [6]. Each starts as a little stub, a neurite, on the cell body of its neuron and that stub has to extend, growing along a specific trajectory until the axon reaches its appropriate target site, sometimes far away at the end of a tortuous path. To find out how the axon grows, we have to look into the machinery at the tip of the axon that drives its extension and allows it to follow its particular path. The heart of this growth machinery are two structural proteins, actin, which polymerizes into filaments to form a dynamic meshwork that generates shape and force, and tubulin, which forms a second kind of structural element, the microtubules, that cooperate with actin to build the axon. The challenge, then, is to understand how the organization and dynamics of actin and microtubules change in response to the local tissue environment to produce guided axon growth. This problem of how axons grow has long been recognized to be one of the central mysteries in neuroscience [7]. Many excellent reviews provide a detailed picture of the different hypotheses that have been offered to account for the mechanism of axon growth and guidance [8–12]. Despite extensive study, however, it has been extremely difficult to integrate these differing ideas into a unified understanding of axon growth and even more so to apply those hypotheses to specific axon patterning decisions that one observes *in vivo*.

Here, we will approach this same fundamental problem, but in a different way. Rather than trying to provide an encyclopaedic overview of investigations into the mechanism of axon growth and guidance, we will restrict our focus to analyse a single, specific axon developing in its native tissue, an axon, moreover, for which the underlying dynamics have been quantified in detail *in vivo* at multiple levels of resolution. After reviewing some of the key data, we will discuss how the observations of this axon, called TSM1 (twin sensillae of the margin 1), suggest a new and unexpected model for the mechanism of axon growth and guidance. In brief, we will see how the stochastic fluctuations of individual actin filaments produce dynamic redistribution of the global actin network in the axon at a multi-micrometre scale and how this in turn produces directed axon growth. To come to that endpoint, we will start by examining the large features of the TSM1 axon and work our way down to the small ones, start with a description of the morphology and actin distribution of this axon *in vivo*, then focus down on biochemical processes that produce that cell biology, and then focus further on the single-molecule biophysics that underpin the entire mechanism. We then synthesize these findings in a coherent model for the mechanism of growth of TSM1. Finally, we show how this model uncovers an underlying simplicity that may reconcile some of the divergent views in the field of axon growth and explain some longstanding mysteries. In the body of this Commentary, we will focus our attention exclusively on the dynamics of the actin cytoskeleton. At the end, however, we will briefly extend our discussion to consider some of the ways that the microtubule cytoskeleton may fit into the story to modulate the actin-based processes that are presented here in more detail [13–15]. It should be kept in mind that throughout this Commentary, we will be presenting our own view of current ideas in the field and the challenges presented by recent *in vivo* experiments. Other investigators will unquestionably view each of these somewhat differently than we do. Our goal, however, is to stimulate discussion and further experiments. If we achieve that effect, then this paper will have fulfilled its purpose. Before we begin, however, we must first give a deeper introduction to the nature of an axon and the events that occur at its growing tip.

## 2. Description of the problem: what is a growth cone and how does it guide axon extension in a developing nervous system?

As mentioned above, when building a nervous system, nearly every neuron extends a long, thin, cytoplasmic process, an axon, to deliver information to functionally downstream elements of its neural circuit. These axons are built using dynamic actin networks, together with arrays of outward-polarized microtubules that provide structural support and tracks for the back-and-forth trafficking of proteins and membrane-bounded vesicles (see [16] for a detailed introduction to the cell biology of the mature axon). Axons can be only micrometres long, or they can extend for metres, depending on the organism. Their morphology can be extremely simple or they can be highly elaborated, with hundreds of branches. In general, however, the path taken by an axon as it grows is distinctive and reproducible for a neuron with a given identity [17]. In the *Drosophila* ventral nerve cord, for example, nearly every one of the 270 neurons found on each side of each segment is individually identifiable based just on the beginning, end and general path of its axon [18]. As any given axon extends, it is bombarded with information from its environment telling it where to grow and where not to grow. Attractant and repellant molecules can be diffusible or membrane-tethered, and signals from a dozen or more such ‘cues’ may need to be summed at any single point in the trajectory of an axon to determine where that axon is going to go [19–21]. How does an axon carry out that morphogenetic calculation, and how are we to dissect such an elaborate process?

The first axon to traverse a particular developmental territory is called a pioneer axon, as opposed to follower axons that approach their target zone by growing along the shaft of a previously established axon [22–24]. The distinction is not just temporal, but rather it harbours functional significance. Ablation of the pioneer axon can lead to stalling, misrouting and failure of follower axons to approach their targets, even if other axon tracts are within the area [25]. Consistent with their specialized function, pioneer axons can demonstrate different growth kinetics and growth cone morphologies than follower axons, as shown, for example, in zebrafish commissural axons [26]. Similarly, experiments employing fluorescence recovery after photobleaching demonstrate slower protein diffusion in pioneer growth cones when compared to followers, perhaps due to differences in actin architecture during active pathfinding [27]. It is the growth and pathfinding behaviour of such pioneers that has most attracted the attention of neuroscientists as they seek to understand how the earliest axons are guided to establish the axon scaffold of the developing nervous system.

**Figure 1.**
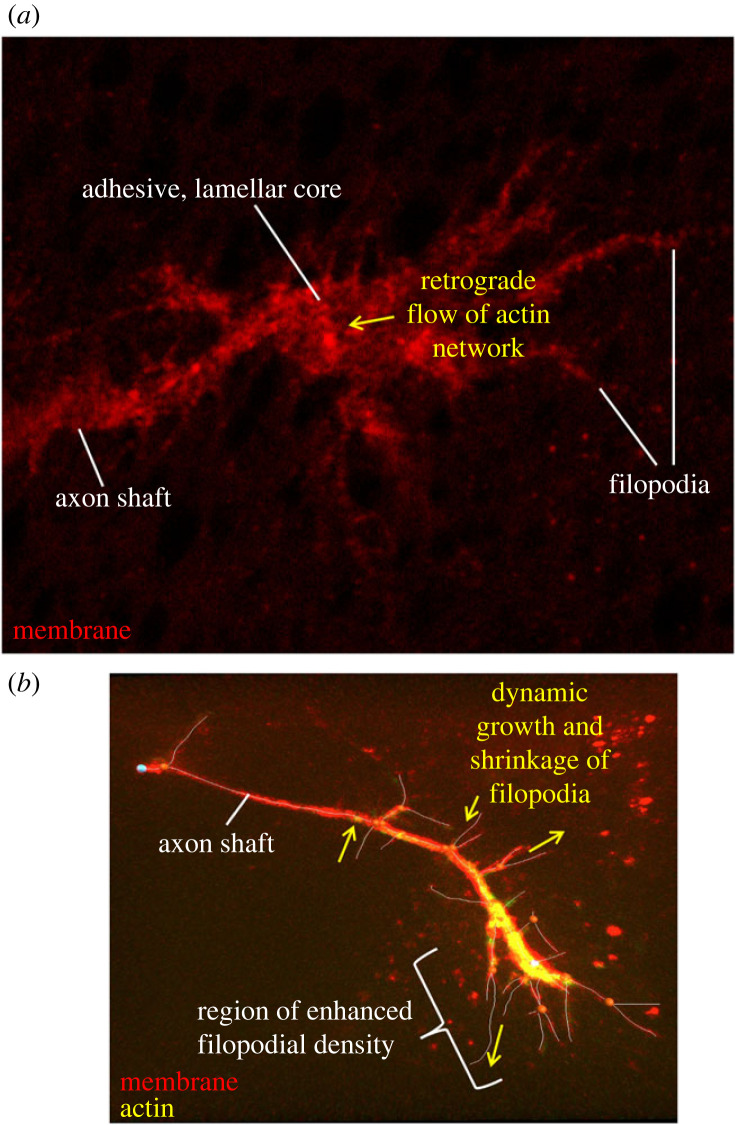
Images of two styles of growth cones. (*a*) A typical adhesive, lamellar growth cone as often seen *in vitro*. The adherent core of the growth cone can be seen in the middle, with axon shaft projecting to the left and filopodia decorating the entire growth cone. Motility is thought to be driven by retrograde flow of actin in the palm of the growth cone, linked to the substratum by transient adhesive contacts to provide points of traction. Note that lamellipodial actin mesh was not visualized experimentally for this micrograph, but its presumed location and motion are represented by the yellow arrow. (*b*) A filopodial TSM1 growth cone. Membrane is labelled in red; actin in yellow. Filopodial projections have been traced (white lines). Note the concentration of actin near the distal end of the axon, associated with the region of highest filopodial density. Motility is associated with dynamic extension and retraction of filopodia (indicated with yellow arrows), with growth cone advance produced by preferential stabilization of filopodia whose orientation correlates with the direction of future axon growth. Note that this image presents a two-dimensional projection of this growth cone. In the animal, filopodia project in three dimensions, spreading as much above and below the plane of imaging as they do side-to-side.

The tip of the pioneer axon is decorated with a ‘growth cone’—a dynamic, exquisitely sensitive domain that surveys the immediate extracellular space for guidance cues and restructures its cytoskeletal architecture to respond and advance, thereby leading the axon that trails behind it [27]. Based primarily on observation of axons growing in culture on rigid surfaces, the growth cone has traditionally been viewed as an adhesive structure, often (though not always [28]) characterized by a broad, flat, lamellar core decorated by a fringe of filopodia, long, finger-like extensions containing approximately 10–20 bundled, parallel actin filaments with their barbed ends at the filopodium tip (figure 1*a*) [29]. Such growth cones are often described as being segmented into three regions: a microtubule-rich central domain, a transition zone and an actin-rich peripheral domain [10]. There are two general classes of mechanisms that have been proposed to explain how such adherent axons navigate and extend. Perhaps the most commonly discussed view is called the ‘adhesion clutch model’ [8,10,30]. This model proposes that polymerization of actin near the front of the growth cone, together with myosin contractility and actin disassembly at the back, produces persistent retrograde flow of the actin network in the growth cone [9,31,32]. Episodic linkage of the actin to cells or extracellular matrix underlying the axon, through cell-surface adhesion proteins, then provides points of traction that pull the axon forward [30,33,34]. An alternative view of adherent axon growth has been called the ‘protrusion, engorgement, consolidation’ or ‘PEC’ model. By this view, it is microtubules that are thought to be essential for advancing the leading edge of the growth cone by some combination of mechanical force and delivery of essential components [8,11,35]. Actin, in contrast, is thought to serve primarily to restrain microtubules and to guide their spatial distribution, not to provide the force for growth cone advance. The evidence for this hypothesis comes in part from experiments demonstrating that disassembly of actin can lead to increased invasion of the most distal part of the growth cone by microtubules and enhanced axon growth [36]. This model derives largely from examination of lamellipodial-dominated growth cones, but evidence for it can also be found in adherent, filopodial-dominated growth cones growing in two-dimensional cultures [28].

While both models discussed above can account for aspects of the axon growth observed in adherent cultures *in vitro*, various experiments have suggested that this picture may not be sufficient to capture the variety of axon growth processes that occur *in vivo*. Enhanced ability to watch axons as they grow in their native tissues, and extension of *in vitro* conditions to the more physiological conditions of a three-dimensional growth matrix and a mechanically compliant environment, has revealed that there is another mode of growth, at least as common and perhaps even more common than the adhesive modes. These are growth cones that seem to move through non-adhesive mechanisms [37–39]. They tend to be characterized by a local profusion of filopodia in the distal part of the axon and have few, if any, lamellipodia (figure 1*b*). The filopodia dynamically survey the three-dimensional volume surrounding the growth cone, and guided axon advance is achieved by selective stabilization of an appropriately oriented filopodium and disassembly of the rest [15,37,40–42]. For pioneer axons *in vivo*, at least, both classical studies and recent observations suggest that such filopodial-dominated, non-lamellar growth cones may be the predominant species [14,38,41,43]. An analogous distinction between adhesive motility in two-dimensional culture and non-adhesive, protrusive motion in three-dimensional media has also been described for the motility of various types of migrating cells [44]. Given that many groups have shown profound effects of differential stiffness and other substrate mechanical properties on neurite outgrowth [45–47] and that *in vitro* cultures have most often relied on adherence to rigid plastic or glass, it perhaps should not be surprising that to understand the mechanism of motility *in vivo* we have to look at axons growing under mechanically soft conditions like those seen in the living animal [48,49]. What, then, is the nature of actin distribution and morphological dynamics in a pioneer axon growing in its native environment, and how do they produce the selective stabilization of particular filopodia that is the basis of guided axon growth? To investigate this question in mechanistic detail, we will next focus our attention on the properties of a single, identified growth cone, in this case, one that is traversing its native trajectory in the developing wing of *Drosophila*.

## 3. Live imaging of a pioneer axon *in situ*: the case of *Drosophila* TSM1

TSM1 is a sensory neuron of the *Drosophila* wing. It is born late in larval development and then, shortly after the onset of metamorphosis, it elaborates an axon that follows a stereotyped, curved path that first projects across the wing, then reorients to grow proximally towards the base of the wing before passing into the thorax to find the central nervous system [50].

**Figure 2.**
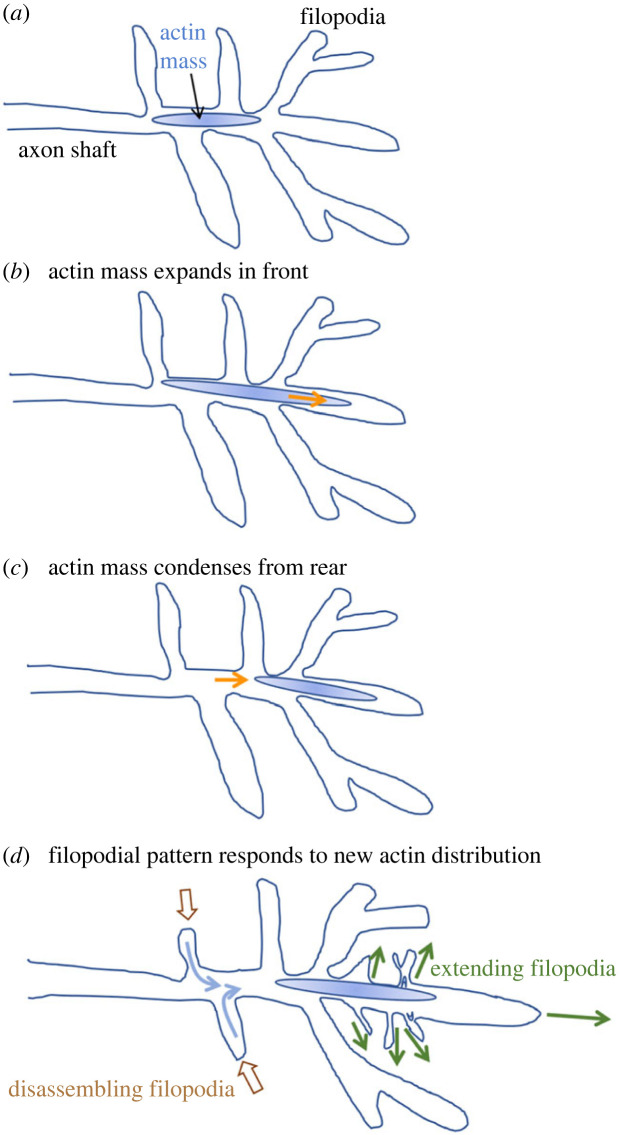
Schematic depiction of the progression of an extending TSM1 growth cone. (*a*) Actin localization in a typical growth cone. For simplicity, the actin mass in the distal axon is indicated as a graded blue oval; see later figures for more detailed depictions of actin organization. The actin mass tends to extend preferentially in the front (as in (*b*); orange arrow) and condense from the rear (*c*), leading to net forward motion of the actin over time. Net motion of actin is followed, after a delay, by equilibration of the filopodial pattern (*d*), with projections tending to disassemble in regions that have lost actin from the axon shaft (brown arrows) and extend in regions that have gained actin density (green arrows).

The structure and dynamics of the TSM1 growth cone were quantified by simultaneous fluorescent live imaging of membrane and actin distribution. The TSM1 growth cone has qualitatively evident lamellipodia in fewer than 1% of imaged timepoints [37,51]. Rather, the growth cone is recognizable as a region in the distal portion of the axon that contains a cluster of filopodia (usually about 20 filopodia), but with no discrete morphological transition between the growth cone area and the axon shaft and only modest growth cone engorgement perpendicular to the axon shaft. The growth cone, however, is invariably highlighted by a high, local accumulation of F-actin (called ‘the actin peak’) in contrast to the low, fairly consistent level of actin intensity filling the remainder of the axon, including all the filopodia (figure 1*b*). The length of the actin peak in the growth cone fluctuates around a median value of about 12–15 µm long, and it is positioned near the front of the axon, associated with the filopodial-rich domain, but is generally not at the very tip of the axon. Note that while, for simplicity, we refer to this concentration of actin as an ‘actin peak’, we presume that it is in fact a contractile structure rich in myosin II as well as actin, in addition to containing a host of other typical actin-associated proteins [52,53]. Between any two timepoints, the actin peak may move forwards, backwards or not move at all. Nonetheless, over the course of minutes to hours, the fluctuations of the actin show a persistent, forward, spatial bias that results in net forward motion of the actin peak as the axon grows (figure 2).

The leading filopodial domain and the associated actin peak, naturally, both advance with time as the axon grows. However, while the positions of the actin peak and the filopodial domain are correlated, they are not coincident. Instead, it is the motion of the actin peak that leads axon growth, and advance of the filopodial domain is a response that follows after. In any given timepoint, the midpoint of the actin-rich region tends to lead the midpoint of the filopodia-rich region by about 3 µm. Given the growth rate of the axon, that means that the position of the actin peak predicts where the filopodial domain will be located about 15 min later. This makes sense intuitively, since filopodia require actin for their growth and maintenance. Therefore, a region of the axon shaft with a high local concentration of actin has the raw materials to make new filopodia. Conversely, a region of the axon shaft with relatively less actin becomes a local sink for actin, promoting local disassembly of filopodia in that region. In other words, the offset between the actin-rich and filopodial-rich domains is a simple consequence of hysteresis, the time required for local filopodial density to equilibrate to the local availability of actin. The essential, leading role of actin dynamics in growth cone advance is also evident from functional experiments. If actin dynamics is inhibited pharmacologically, for example, growth cone advance stops immediately. Together with other observations [37], these data argue that the pulsatile advance of the actin mass is what drives extension of the axon, with morphological advance being a downstream response.

In summary, the TSM1 growth cone displays an ‘inchworming’ style of protrusive axon growth. The actin peak expands and contracts along the direction of axon advance. Due to preferential extension at the leading edge and compaction at the trailing edge, the actin mass moves distally. The resulting relocation of actin spurs new filopodial growth further distally and filopodial retraction behind. This continuous process causes processive axon growth, and blocking the fluctuations of actin stops axon growth. The next question, therefore, is what controls the distribution and dynamics of actin.

## 4. The Abl tyrosine kinase pathway and regulation of actin organization

Since the analysis of TSM1 finds actin dynamics to be a critical driver of axon growth, we next need to consider the signalling mechanisms that coordinate the elementary steps of actin dynamics—actin nucleation, polymerization, branching and disassembly [54]. A uniquely effective place to begin is with the Abelson non-receptor protein tyrosine kinase (Abl), a phylogenetically conserved protein that acts downstream of many of the conserved families of growth cone receptors, including the receptors for netrins, ephrins, slits, semaphorins, integrins and others [55–61], but upstream of many regulators of elementary steps in actin dynamics, including cofilin, Arp2/3 (a complex containing actin-related proteins 2 and 3), Ena/VASP proteins (enabled/vasodilator-stimulated phosphoprotein) and myosin II [62–66]. Abl is therefore an exceptionally good candidate to integrate the signals coming from a multiplicity of external guidance cues and to link the complex extracellular signalling milieu to intracellular cytoskeletal reorganization.

**Figure 3.**
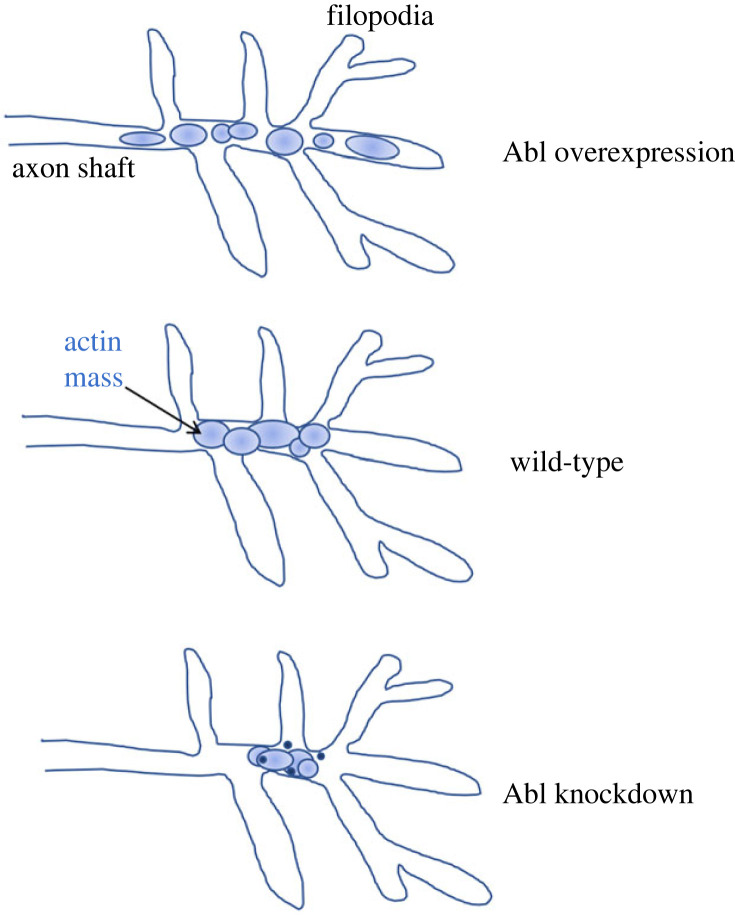
Schematic depiction of the effect of altered Abl kinase activity on the distribution of actin in TSM1. Organization of growth cone actin into domains (highly cross-linked subnetworks; see text) is indicated by separated ovals (compare figure 1*b*). In Abl overexpression, these tend to spread out over a larger region; in Abl knockdown they tend to condense, and often form dense puncta.

It has been shown in many systems, including TSM1, that Abl is a critical component of the signalling network that controls growth cone actin organization. Either genetic knockdown or overexpression of Abl leads to significant axon stalling and misrouting, terminal phenotypes that indicate a failure of axon growth and/or guidance [67–71]. In wild-type TSM1 axons, the length of the actin peak changes over time as the actin distribution fluctuates at its leading and trailing edges, and Abl turns out to be a key regulator of this distribution: Abl knockdown decreases the average actin peak length, and Abl overexpression increases it [37]. The effect of Abl on actin distribution can also be seen in properties that are more subtle, but that turn out to have profound effects on function. If one analyses the actin distribution at higher resolution, one sees that reducing the level of Abl causes actin to hypercondense into high-density blobs, and in concert with that condensation, the actin fluctuations that are the motor for axon advance are attenuated. Conversely, overexpressing Abl causes the actin peak to fragment, spreading out on a scale of multiple micrometres into partially disconnected subdomains and apparently reducing the ability of the actin network to act as a single, concerted unit [51,72]. These observations help explain an old conundrum. Gain or loss of Abl activity produces superficially similar axon defects, axon stalling and misrouting. We now see, however, that mechanistically these arise from opposite sources, hypercondensation of actin in the case of Abl loss-of-function, versus spreading and fragmentation of the network in the gain-of-function. Both perturbations interrupt the orderly, pulsatile fluctuation of the growth cone actin network that is required to produce forward motion, but the effects on actin distribution are opposite and (as we will see below) arise from opposite biophysical processes (figure 3).

In addition to modifying actin organization in TSM1, Abl also modifies growth cone morphology. For example, the number and total length of filopodia are altered in Abl knockdown and overexpression [37]. However, these morphological effects are far more modest than are the effects on actin organization. For example, one can use principal component analysis (PCA) to construct a quantitative composite measure that integrates multiple aspects of actin distribution (actin peak length, fragmentation, dynamic instability and offset from the morphological centre of the growth cone) and compare it to an analogous composite measure of filopodial parameters (filopodial number, length, branching complexity and volume encompassed by the filopodial network). This comparison shows that the effects on actin organization from altering Abl activity are approximately 10 times as great as the effects on morphological features of the growth cone. This further underscores the primacy of actin organization as the direct target of growth cone signalling activity, with morphology being a secondary, emergent property. It also emphasizes the need to focus-in further on the mechanisms connecting cytoplasmic signalling to actin dynamics if we are to understand growth and guidance of TSM1 [9,73,74].

## 5. The organization of the Abl signalling network

Abl, therefore, acting in response to external cues, is essential and instructive for actin distribution and dynamics in the TSM1 growth cone, but what is the mechanism? Though Abl does have an F-actin binding site in its C-terminal domain [75], this does not appear to be essential to its axon patterning function [76,77]. There must, therefore, be other downstream molecules between Abl and changes in actin structure and axon growth. Abl links to a number of signalling proteins that impinge on the actin cytoskeleton of the axon, but genetic experiments in *Drosophila* highlight two in particular. The Abl network bifurcates to control two, parallel, actin regulatory mechanisms, the linear actin polymerase, Enabled (Ena) and a Rac (Ras-related C3 botulinum toxin substrate) signalling pathway that regulate the WAVE (WASP-family verprolin-homologous protein) complex and thus the branching actin nucleator, Arp2/3 [64,76,78].

**Figure 4.**
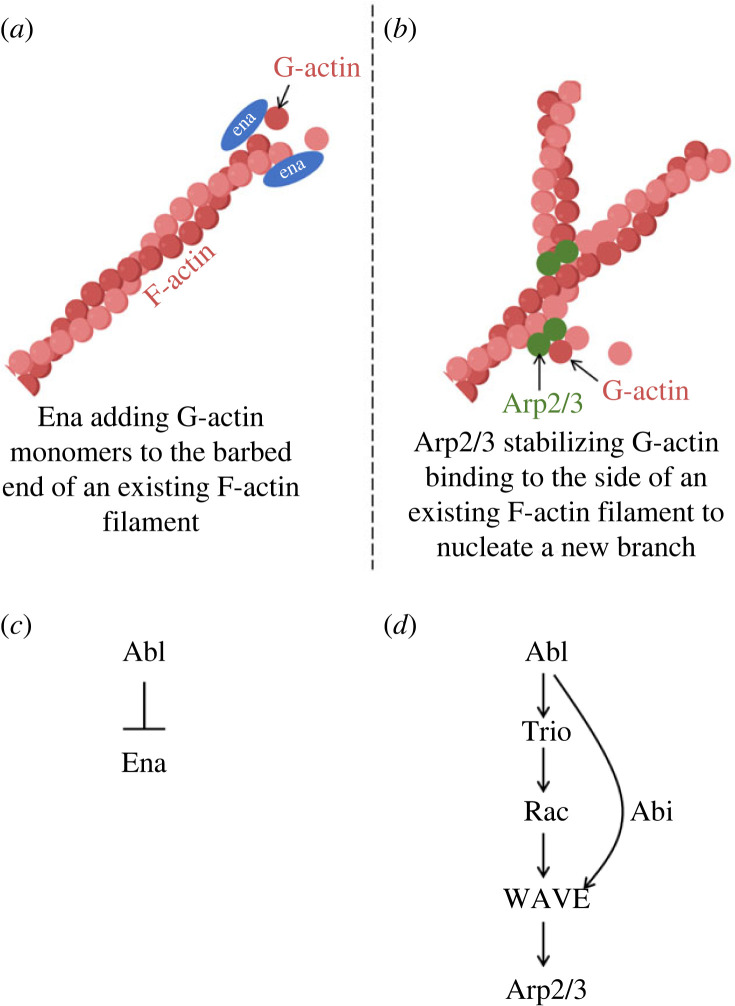
Schematic depiction of actin polymerization and nucleation by Ena and Arp2/3, respectively, and the relationship of Abl to the activity of each. (*a*) Ena (blue oval) has both an F-actin and a G-actin binding domain and stimulates actin polymerization in part by transferring a G-actin monomer (red circle) to the barbed end of an existing F-actin filament (depicted as a helix of G-actin monomers). Ena is present *in vivo* as a tetramer, but for clarity only two monomers of Ena are shown here. (*b*) Arp2/3 (green circles) nucleates initiation of a new actin filament by binding to the side of an existing filament and providing a site for stable binding of G-actin monomers from solution. Additional monomers can then add to the end of the newly formed filament. (*c*) Abl negatively regulates Ena activity. This occurs by at least two mechanisms, direct phosphorylation of tyrosine residues on Ena, which is apparently inhibitory for Ena function, and also regulation of Ena localization that excludes Ena from particular regions of the cell. The mechanism by which Abl controls Ena localization is not known. (*d*) Abl positively regulates Arp2/3 activity. Abl activates the Rac-specific GEF Trio and Rac in turn activates WAVE complex. WAVE then recruits Arp2/3 and stimulates its actin nucleation activity. In parallel, Abl also associates physically with WAVE complex components, notably Abi (depicted as an additional arrow in the diagram).

Ena is perhaps the best characterized downstream target of Abl (figure 4*a*,*c*). It acts as a processive linear actin polymerase and a bundler of actin filament tips [79,80], and it prevents capping of the fast-growing ‘barbed’ end of an actin filament. Abl kinase phosphorylates tyrosine residues on Ena [81], and this phosphorylation modifies the activity of Ena in *Drosophila* and of its paralogues in mammals (Mena, Evl and VASP) [82]. However, direct Abl-mediated phosphorylation cannot fully explain the regulation of Ena, as expression of a non-phosphorylatable Ena derivative rescues many of the phenotypes of an *ena* mutant [83]. Another key element of Ena regulation by Abl is at the level of subcellular localization. In the early *Drosophila* embryo, for example, much of the mutant phenotype in animals lacking Abl arises from ectopic accumulation of Ena protein at cell cortices, with consequent ectopic assembly of actin and formation of aberrant actin structures [64]. The mechanism by which Abl normally excludes Ena from such inappropriate localization remains obscure, however.

A second major pathway by which Abl promotes actin assembly in *Drosophila* axons is via the guanine nucleotide exchange factor (GEF) Trio [84] (figure 4*b*,*d*). Trio is a large protein containing two GEF domains, one specific for Rac GTPase and the other specific for Rho. Of these, only the Rac-specific GEF domain is essential for the axonal processes so far shown to require Trio in vertebrates and invertebrates [85–87]. In the absence of Abl, the Rac GEF domain of Trio is repressed by an intramolecular inhibitory interaction with an N-terminal spectrin repeat domain of the protein [78,88], but this repression is relieved by Abl through a mechanism requiring Abl kinase activity (though the direct target of Abl phosphorylation in this process has not been identified). Activation of Trio, in turn, allows it to stimulate the signalling activity of Rac GTPase. Rac then associates with and activates the WAVE complex, which promotes initiation of new actin filaments by inducing the actin nucleating factor Arp2/3 to bind to the sides of existing actin filaments and accelerate the rate-limiting first step of actin assembly [89,90]. Consistent with this overall picture, extensive genetic experiments have demonstrated that Trio is a core component of the Abl signalling network: mutants of *trio* display many of the same axonal phenotypes as *Abl*, and mutations in these two genes interact synergistically in those processes [84,87]. Similarly, Abl has been shown to interact genetically and biochemically with various components of the WAVE complex, including Abi-1, a WAVE component that was first identified as a protein that physically associates with Abl [76,84,91,92]. The biochemistry of these two key Abl targets, Ena and the Trio–Rac–WAVE pathway, is therefore reasonably well understood. Curiously, however, Abl regulates these two effectors in antagonistic ways, suppressing Ena activity but stimulating Trio–Rac–WAVE [78]. Evidently, modifying Abl activity changes the balance between these two effector pathways, with increasing Abl favouring branching nucleation and decreasing Abl limiting such nucleation. This still leaves a puzzle, however. The actin cytoskeleton is a complex, three-dimensional structure that spans many micrometres. How do the modifications produced by Abl effectors working on one actin filament at a time cause multi-micrometre-scale reorganization of the overall actin cytoskeleton to produce directed axon growth?

## 6. Computational simulations suggest how the dynamics of single actin filaments produce the large-scale structure of an actin network

The ideal experiment to link single-filament actin dynamics to the multi micrometre-scale organization of the axonal actin network, of course, would be growth cone live imaging at single-molecule resolution. However, there is no existing form of microscopy that even comes close to offering simultaneously both the spatial and temporal resolution required to resolve the dynamics of single actin filaments deep inside a developing tissue. On the other hand, we know a great deal about the detailed biochemistry of actin and its associated regulatory proteins. This offers the opportunity instead to dissect the mechanism and consequences of Abl-dependent actin reorganization by performing computational simulations of actin dynamics [93], manipulating the actin-interacting proteins we know to be the key players downstream of Abl itself. These studies reveal how the interplay between myosin contractility and the distribution of the lengths of actin filaments tunes both the mesoscopic dimensions of the actin network and the spatial range over which force can be transmitted within that network.

The experiment performed *in* *silico* was to generate a cylindrical volume the size of a TSM1 growth cone (2 μm in diameter and either 7.5 or 15 μm in length), fill it with G-actin, myosin II an actin cross-linker (α-actinin) and varying levels of the key Abl effectors, Ena and Arp2/3. The ‘growth cone’ was then allowed to undergo dynamics for 2000 s (long enough to achieve steady state), incorporating both chemical events, such as polymerization, branching, bundling and contractility, as well as relaxation of physical strain every 25 ms. Kinetic constants for the simulations were taken from published values for the relevant dynamic processes, and concentration ranges of components were selected based on a combination of theoretical considerations and empirical tests [52,94]. While extraordinarily rich in detail, the resulting findings were remarkably simple in terms of the principles that they revealed.

As expected, under the influence of Ena and Arp2/3, G-actin monomers in the simulation volume polymerize into F-actin filaments. Moreover, the concentration of these two key regulators in any given simulation was the main factor that determined the distribution of lengths of the actin filaments that were present when that simulation reached steady state. What was not expected, however, is that the differences in those final, steady-state distributions of actin filament lengths is the key parameter that determines the overall, multi-micrometre scale organization of the final actin networks, as described below.

**Figure 5.**
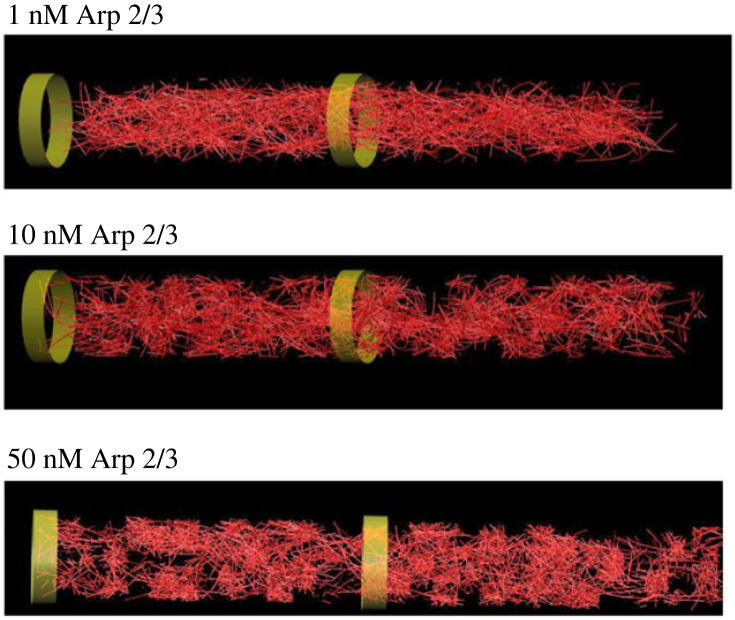
Final snapshots from computational simulations at low, medium and high levels of Arp2/3 (modelling low, medium and high levels of Abl kinase activity). Red lines indicate actin filaments. Green bands indicate the dimensions of the simulation volume. Note condensation of actin mass at low Arp2/3, as opposed to actin dispersion at high Arp2/3 with development of voids in the midst of the actin domain. Intermediate Arp2/3 gives an intermediate distribution, more homogeneous than that at high Arp2/3 but less condensed than that at low Arp2/3. Simulation volume was 15 µm long and 2 µm in diameter.

**Figure 6.**
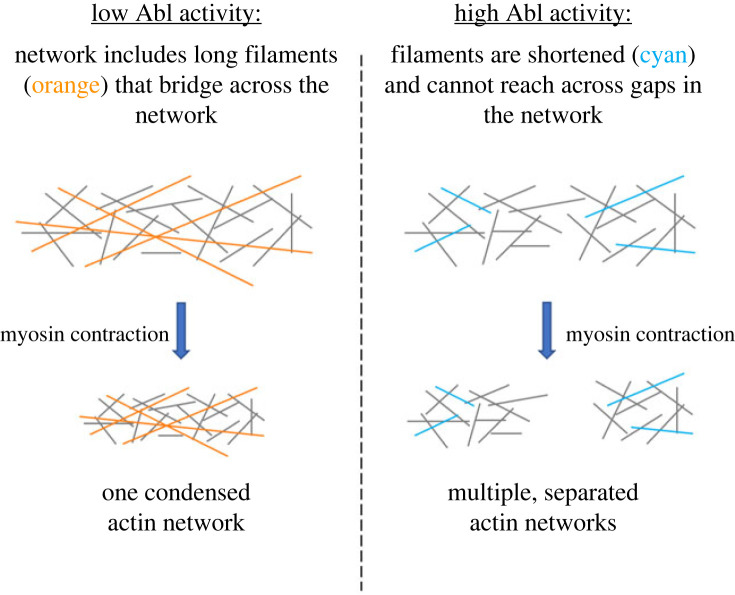
Schematic depiction of actin network organization at low versus high Abl activity. At low Abl activity (low Arp2/3), the actin network includes a population of long filaments (orange). Since these can bridge between subnetworks, applying myosin contractility causes the entire network to condense as a single unit. At high Abl activity (high Arp2/3), enhanced nucleation shifts the actin filament distribution towards shorter lengths (blue filaments). In the absence of a population of long filaments, contractility leads to local condensation of individual actomyosin subnetworks but the subnetworks remain separate and spread out through the reaction volume. Actin filaments are shown as lines; for clarity, myosin is not shown.

Like many stochastic networks, actin self-organizes into a ‘small-worlds’ structure. That is, while most individual actin filaments in the network have cross-links to at least one other filament (provided in the form of Arp2/3-linked branches, or by simultaneous binding to the same two-headed myosin motor or the same molecule of α-actinin cross-linker), the network tends to develop such that there are local regions (subnetworks) within which those cross-links are quite dense, but that separate subnetworks are connected to one another by only a much sparser set of cross-links (figure 5) [52]. The spatial scale of these actin networks and subnetworks, however, depends critically on the distribution of lengths of the individual actin filaments (figures 5 and 6). When the concentration of actin nucleator is low (low Arp2/3, as one would see at low Abl kinase activity), actin turnover maintains a substantial fraction of long actin filaments, in the range of 1–2 µm in length. In this context, long filaments can bridge between neighbouring subnetworks. Upon application of myosin contractility, therefore, force is transmitted between subnetworks, across the entire simulation volume. Thus, the entire collection of actin subnetworks condenses as a single, dense unit in the centre of the reaction volume. In striking contrast, however, if the concentration of nucleator is raised (high Arp2/3, as one would see at high Abl kinase activity), it causes the same amount of actin to be broken down into more filaments, causing each filament to be shorter (mode filament length approx. 0.25 µm). In this case, while actin still self-assembles into subnetworks, there are few filaments long enough to bridge between those subnetworks. Consequently, upon application of myosin contractility, each subnetwork condenses locally, but the subnetworks are mechanically disconnected from one another and they spread apart to distribute, separately, across the whole simulation volume. In short, extension of single filaments causes the overall actin network to shrink under contractile force, whereas shrinkage of the individual filaments causes the network as a whole to expand.

The pattern of overall compaction versus expansion of the total actin network with changing Arp2/3 activity that was observed in the simulations closely matches what was observed experimentally from loss and gain of Abl activity in TSM1 growth cones [37,51] and of Arp2/3 in VD motoneuron growth cones of *Caenorhabditis elegans* [95]. Moreover, the hypercondensation of actin into dense foci that was observed experimentally from reduction of Abl in TSM1 and the splitting of actin density into dispersed domains upon increase of Abl were also mirrored in the computational output. These observations therefore provide strong evidence that the computational studies capture essential aspects of the mechanism by which manipulation of simple, elementary processes of single-filament actin dynamics propagates to produce the multi-micrometre scale changes in the dimensions of actomyosin networks that one observes in TSM1. The community structure of the actin network, and its striking dependence on filament length distribution, has another consequence with potentially far-reaching functional implications. The larger, more coherent actin networks that form in the presence of a modest proportion of long filaments are also capable of transmitting force over long distances across those networks, whereas the fragmented, mechanically disconnected networks generated with short filaments are not capable of force transmission across a multi-micrometre scale [52]. This provides a physical basis for the observation that increase of Abl activity expands the actin network beyond the range at which mechanical information (i.e. force) from one end of the TSM1 growth cone can be transmitted to the other end [51,72]. It also suggests that filament length-dependent network fragmentation could, in principle, be exploited to modulate the spatial range of force transmission across cells in a wide variety of cell biological processes.

The analysis above focuses on Arp2/3, but what about the other branch of Abl signalling, via Ena? Surprisingly, simulations suggest that the effects of Ena on large-scale actin organization should be minimal (Ena can only extend the ends of existing, uncapped actin filaments, which are few in number, whereas Arp2/3 can nucleate a new filament essentially anywhere along the side of an existing filament). This was unexpected, but direct experimental quantification of actin distribution in TSM1 upon decrease or increase of Ena activity validates the prediction, demonstrating that altering Ena activity does not produce statistically significant effects on actin distribution in the growth cone. Instead, the key effect of Ena is evidently on the linkage of that actin to the plasma membrane, that is, on the efficiency with which filopodia are produced in response to accumulation of actin. It seems that the complementarity of Abl effects on Ena versus Arp2/3 is likely a mechanism to ensure that growth cone morphology remains stable even as Abl activity is altered to vary the properties of the growth cone actin network [39]. It is interesting that in other biological contexts, Ena, as well as its vertebrate orthologues, is evidently capable of having more pronounced effects on actin organization than is seen in the TSM1 growth cone [96–98]. The basis for this is unclear, but it may be related to the fact that the studies demonstrating stronger direct effects of Ena largely analyse adhesion-limited processes, as opposed to the protrusive process of TSM1 extension. Further study will be required to assess this conjecture.

## 7. Synthesis: how the stochastic dynamics of actin networks may account for axon growth and guidance *in vivo*

Can we, then, bring together cell biology, biochemistry and biophysics to account for the guided growth of the TSM1 pioneer axon *in vivo*? Can we do so in a way that is true to the inherently probabilistic nature of single-cell biology, as reflected in the choices made by an individual pioneer growth cone? And what are the implications of variability and stochasticity for the mechanism, and the fidelity, of neural wiring?

We have seen here that the morphological feature that we call ‘the TSM1 growth cone’, a local domain of dynamic filopodia, is an emergent property generated by the presence of a local accumulation of actin in the axon shaft. The actin network in the axon fluctuates stochastically in length, but a small spatial bias favours fluctuations that advance the actin along its genetically pre-specified trajectory. The actin mass as a whole thus advances, and in so doing, it advances the morphological domain that is competent to support filopodial formation and maintenance, i.e. the growth cone. In essence, net advance occurs by locking-in those stochastic fluctuations of actin that happen to occur in a direction favoured by external, environmental factors, in this case guidance cues laid out by the genetic programme of axon wiring, together with the physico-chemical properties of the underlying substratum. In this picture, for clarity of exposition, we emphasize the actin in the core of the axon shaft, but in reality, that central actin mass is continuous with the actin network throughout the axon, including the filopodia, and the global fluctuations of the entire actin network contribute to the growth and guidance event.

The dimensions and cohesion of the actin network, in turn, are controlled by cytoplasmic signalling pathways such as that defined by Abl tyrosine kinase, among many others. At any given time and place, those signalling pathways integrate information from a complex constellation of chemical guidance cues and receptors, as well as physical features of the internal and external environment, transforming those spatial cues into patterned intracellular modulation of a few elementary processes of actin dynamics—actin polymerization, branching, severing, bundling and capping, together with myosin contractility. Thus, the summed effects of cytoplasmic signalling pathways produce spatial modulation of the detailed kinetic parameters that control actin dynamics. In response to that patterned modulation, the quantitative logic of actomyosin biophysics, and particularly the interplay between the distribution of actin filament lengths and myosin contractility, probabilistically modifies the dynamic architecture of the actin network to promote net forward translocation of the actin mass. In particular, we propose that preferential shortening of actin filaments in the leading part of the actin peak locally fragments the actin mass into smaller subnetworks. This favours local expansion of the actin mass, causing its leading edge to advance. Meanwhile, in the rear of the growth cone, preferential lengthening of individual filaments and/or enhanced myosin II contractility locally promotes consolidation of that portion of the actin mass, effectively compacting it into a cohesive unit that, in the process, pulls itself forward towards the centre of the growth cone. It is the combination of expansion in the front and compaction from the rear that produces the net forward motion of the actin. By this model, in other words, the spatial bias in the fluctuations of actin network density is what drives translocation of the axonal actin peak into a single leading filopodium, transforming that filopodium into the future axon shaft and thus advancing the effective tip of the axon. It is noteworthy that this model does not require formation of specific anchorage points in the environment to provide foci for traction [10]. It was demonstrated many years ago by Purcell that a structure of the physical scale of the axonal growth cone intrinsically operates in a physical regime termed ‘low Reynolds number’, where the effective viscosity of the medium is high and recoil is nonexistent. In this condition, motility can be driven simply by rearrangement of internal components without any need to invoke external traction points [99].

We note that the very same mechanism that extends the axon can also account for growth cone turning. The axonal actin network fluctuates not just along the axon shaft but also out the flanking filopodia. Locking-in fluctuations that preferentially populate an oblique filopodium, rather than a leading one, make that oblique projection the focus of future projection outgrowth—the new growth cone—and the axon has now turned. Note also that for turning of TSM1, as for its growth, there need not be a unique site at which a deterministic, instructive turning signal is received. Rather, guidance information can be distributed over the entire growth cone, and averaged over time and space, to progressively promote the guided advance of the axon.

The key property of the view presented here is that it is irreducibly probabilistic, at every step in the mechanism. Motion of the growth cone derives from stochastic fluctuations of the actin distribution, both in terms of its net position and of its mesoscopic level of fragmentation. That fluctuating fragmentation, in turn, arises from the stochastic properties of actin turnover. Efforts to account for these data by a deterministic model foundered in all cases on the probabilistic behaviour that was observed at every stage of the experimental and computational analyses of TSM1. It has come to be recognized in many systems that cytoskeletal cell biology and biophysics are in their essence inherently probabilistic in nature, not just noisy to measure, but noisy in their essence, and indeed have evolved to use ‘noise’—random fluctuation—as a central element in their mechanism, just as we see here [42,100–103]. In a similar way, noisy fluctuation of filopodial number has been shown to be an essential element in the directional response of growth cones to a netrin gradient in *C. elegans* [42], and stochastic stabilization of a subset of filopodia by microtubule invasion is central to target selection in the *Drosophila* optic system [104]. Single-cell biological mechanisms, including pathfinding of individual growth cones, often cannot readily be accounted for by deterministic models.

The model of growth cone mechanism described above, built on the stochastic fluctuations of actomyosin networks, sounds very different from the typical view of how axons grow [8,10–12]. And yet, that existing view derives from extensive experiments over a span of more than three decades. What is the relationship among these varying views of growth cone function? It has been suggested that there may be a categorical distinction between an adhesive, lamellipodial mode of axon growth on rigid, two-dimensional substrata versus a non-adhesive, amoeboid mode of filopodial-dominated motility in three-dimensional media [38]. However, non-lamellar, filopodial morphology is also seen frequently during adherent two-dimensional growth [28] and lamellipodial morphology can sometimes be observed in growth cones live-imaged *in vivo* [95,105]. Evidence from Dumoulin *et al.* [105] may offer a way to reconcile these views by suggesting that different modes of growth cone function may best be thought of as points along a continuum, with growth cones capable of transitioning between modes depending on internal and external conditions. Thus, growth cones of chicken commissural axons imaged *in vivo* appear as protrusive, filopodial structures when growing laterally across the midline or longitudinally after exiting the floor plate but transiently assume a lamellar structure when they stop moving at the floor plate exit point to reorient their polarity [105]. In this connection, it is interesting to note that the ‘classic’ lamellipodial *Aplysia* bag cell growth cone that has been the primary paradigm for adhesive growth cones [30] is actually a transient, largely non-motile repair state that forms after axotomy, not a rapidly advancing motile structure [106,107]. Stasis and reorientation, moreover, are not the only influences that may favour transitions to a lamellipodial morphology or adhesive behaviour. It is likely, for example, that a relatively rigid or highly adherent substrate, as might be found on a basement membrane, would also be capable of promoting adhesive, lamellar growth. Moreover, it is intriguing to speculate that the adhesive mode(s) of growth might plausibly be used by follower axons to extend along a previously established pioneer tract. Finally, it is also clear that varying growth cone signalling, for example by introducing mutations or specific culture conditions, can induce transitions between lamellipodial and filopodial modes [105] (R. Forghani and E. Giniger 2023, unpublished data).

Along the continuum of growth cone modes, it appears that the model we present here of probabilistic actin reorganization *in vivo* represents yet a third paradigm, one distinct from both of the adherent mechanisms described earlier. It is clearly different from the traction-based mode of adhesive growth first proposed for lamellipodial growth cones as it lacks the structural features on which that mode is predicated. However, it also appears to be distinct from the PEC mode of adherent, two-dimensional motility. PEC, like TSM1 probabilistic actin reorganization, posits that net motion is generated by reorganization of internal components, not by pulling on external traction points. PEC-associated growth, however, is thought to be driven by microtubules invading the leading edge of the growth cone to promote membrane advance, while the role of actin is to limit invasion by microtubules and guide their spatial distribution [13,35]. In this mode, therefore, disassembly of actin unleashes enhanced extension of the axon *in vitro* [11,28]. By contrast, in the TSM1-like mode of growth *in vivo*, the fluctuation and spatial redistribution of actin is itself the driver of axon advance and turning: inhibiting actin dynamics halts axon growth immediately [51]. This is reminiscent of axons in compliant three-dimensional cultures, which also fail to show enhanced extension upon depolymerization of actin [38]. Moreover, while there is strong correlation between the changing distribution of actin and growth of the axon in TSM1, there is no detectable correlation between fluctuations in the position of the leading growth cone membrane and the instantaneous rate of axon extension in this neuron [37]. These properties suggest fundamental differences between the PEC mechanism of axon extension in adherent cultures *in vitro* versus the mechanism of TSM1-like extension *in vivo*. Nonetheless, it remains possible that elements of the TSM1-like mechanism might also contribute in the PEC context. For example, in an adherent environment that predisposes to the PEC mechanism, one could plausibly imagine that the initiating protrusive event could be driven by stochastic actin rearrangement like that seen in TSM1, separate from later steps in the motility cycle. To determine the relationship between the TSM1 mode of *in vivo* growth and the more familiar modes of two-dimensional motility *in vitro* it will be essential to perform equivalent experiments and analyses in the different experimental contexts so that direct comparisons can be made. The morphology observed for TSM1 matches well with those seen in live imaging studies performed *in vivo* in several organisms [15,40,105,108,109], as well as in a soft, three-dimensional matrix in culture [38], suggesting that the TSM1-like mechanism is apt to be widespread in development and phylogeny. Morphology alone, however, may not be sufficient to discriminate among modes of motility without deeper mechanistic investigation.

The three general models of axon growth also differ in how they incorporate the role of force in growth cone advance. More purely adhesive models emphasize the importance of external traction forces for physically pulling the growth cone forward [30]. PEC-style models, in contrast, implicate physical forces primarily in modulating the activity and localization of intracellular signalling complexes in the growth cone [110]. Different from both of these, the probabilistic actin reorganization model described here consigns force to an altogether subsidiary role and instead ascribes mechanism almost entirely to the spatial distribution of chemical signalling. Indeed, periodic dissipation of accumulated forces is an explicit step in the computational simulations of actin dynamics in TSM1 [52,93,94]. Here again, however, it may be that the distinction between these growth cone modes is one of degree rather than being categorical, with these modes defining a continuum of mechanisms. Depending on the external and internal environment, it is easy to imagine that a growth cone may transition between growth modes, or exist in an intermediate state. For example, it seems plausible that a PEC-style growth cone in which force acts primarily to regulate signalling complexes may also apply some of that force to generate external traction should appropriate adhesive contacts develop. Similarly, a highly adherent, lamellar growth cone that encounters a mechanically softer environment of limited adhesivity could presumably reorganize towards an amoeboid style of motion exploiting probabilistic actin reorganization. The existence of distinguishable growth modes in limiting environments does not preclude intermediate mechanisms with elements of more than one mode.

## 8. Generality and functional implications of the TSM1 mode of axon growth

The mechanism we describe has been inferred based on the signalling properties of a single signalling pathway, defined by Abl tyrosine kinase. But how does a growth cone integrate and respond to the complex cocktail of biochemical signals and biophysical constraints provided by the *in vivo* environment? The mechanism that we have described reveals an unforeseen simplicity beneath this apparent complexity. The number of external influences and internal signalling components is vast, but all of that complexity converges on just half a dozen elementary steps in actomyosin dynamics. The outcome in any individual case will depend on the specific nature of the many contributing factors, but they all converge on the same machinery, one responding, in part, to the interaction between the distribution of lengths of the individual actin filaments and the contractility of myosin. That interaction is modified by ancillary processes such as stabilization, bundling, and capping of filaments, but these fit into the picture in ways that also can readily be understood. In detail, each contributing factor will have its own special features—polymerization of actin by Ena/VASP proteins [80] will have different specific properties from that by other actin polymerases, such as formins [111]; severing and debranching of actin by the actin accessory protein, cofilin, will have particular effects on the structural and kinetic properties of the actin network [112]; and these will be different in detail from the consequences of other actin severing proteins, such as the actin oxidizing protein MICAL [113]—but the fundamental effects of such factors on the network organization of actin and its spatial properties of force transmission should be predictable based on the network properties described here. We suggest, therefore, that the principles we discuss should provide a perspective that is generally applicable to many different signalling pathways, and many different direct cytoskeletal effectors, that link to the actomyosin cytoskeleton in a wide variety of growth cone guidance decisions.

The saltatory, episodic nature of axon advance that we have described has crucial implications for the specificity and fidelity of guidance. For example, we have long wondered how a growth cone can navigate in response to the vanishingly shallow gradients of guidance information present *in vivo* [114]. The mechanism we observe here now offers a simple answer. If we add-up the pulsatile fluctuations of actin that occur in the time it takes the TSM1 axon to extend by a distance equal to the average length of this growth cone (approx. 15 μm), such a single step forward takes about an hour and spans more than 250 distinguishable spatial fluctuations of the actin distribution. In other words, during the process of taking a single step forward, TSM makes something in excess of 250 effective measurements of the distribution of guidance cues, spread over a volume that averages approximately 2500 μm^3^ at any given instant. Suddenly, the statistical fluctuations of guidance cues and signalling pathway activity become tools of guidance rather than problems. The growth cone simply averages over time and space, reinvestigating the local concentration of guidance signals in any single voxel many, many times as it slowly advances along its trajectory. This allows accurate and precise measurement of even extremely small differences in the concentrations of guiding molecules. Variability and fluctuation, therefore, are the very essence of the mechanism of growth and guidance of TSM1. They are not ‘bugs’ in the machinery; they are the machinery [115–117]. It may seem that this mechanism is energetically wasteful, in that the net motion of the actin peak is only of the order of approximately 1–5% of the total motion of that peak [37]. We suggest, however, that the energy expended is the cost of specificity in axon guidance, allowing accurate outcomes to arise from the repeated measurement of very small differences. The same principle of spending energy to achieve specificity is found throughout molecular and cell biology, including fidelity in DNA replication and protein translation, the role of apoptosis in synaptic matching, and the common observation of futile cycles in metabolism [118–120]. Finally, here we base our analysis on TSM1, but how local or distributed the spatial signals are in different cases will differ depending on the distribution of guidance cues, receptors, and signal transduction molecules for the growth cone under investigation (see below). Nonetheless, we suggest that extensive spatial and temporal averaging of information at one or more steps in the cascade of signal collection and transmission will turn out to be a common feature in axon growth and guidance.

**Figure 7.**
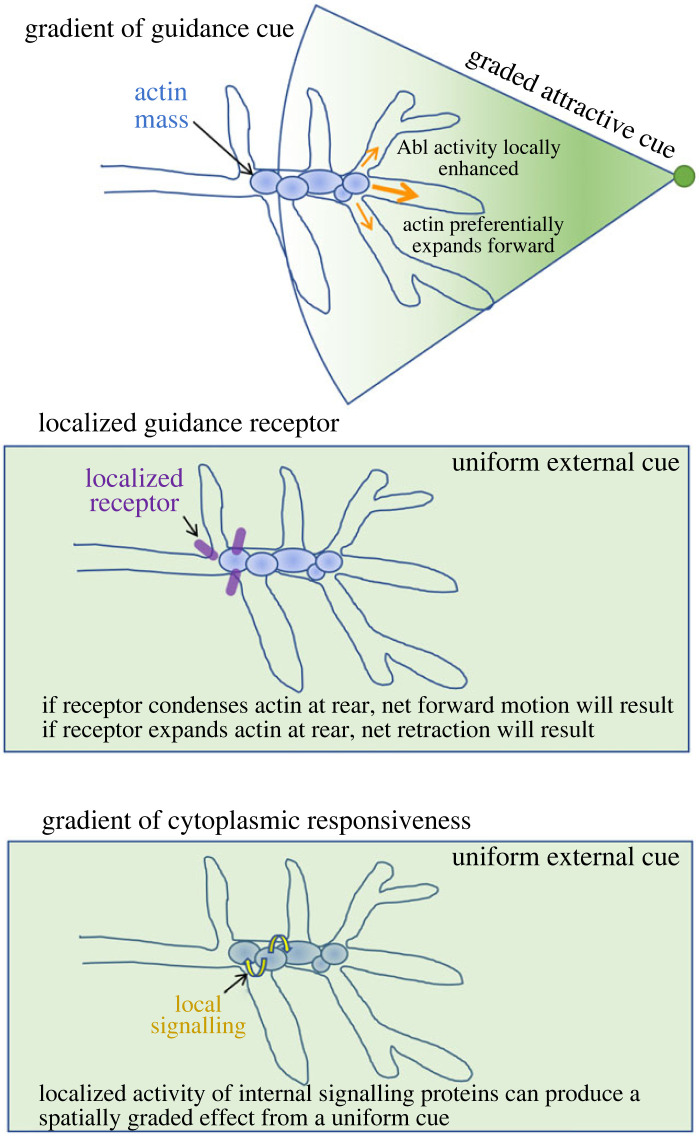
Speculative model for some of the ways that signalling might give rise to spatially graded actin expansion and condensation to promote axon growth and guidance. (*a*) Graded presentation of a guidance cue (depicted as a green shaded cone), whether diffusible or surface-bound, could, in principle, give rise to spatially graded activity of Arp2/3 and other actin regulatory factors to promote preferential actin expansion in the required direction (orange arrows). (*b*) Even in the presence of a uniform guidance cue (green box), localization of the cognate receptor (purple oblong) to just one end of the growth cone could preferentially promote expansion or condensation of actin at that end, thereby leading to net growth or net retraction of the axon, depending on the nature of the biochemistry. (*c*) Spatially inhomogeneous feedbacks in the cytoplasmic signalling network (yellow, curved arrows), localized distribution of cytoplasmic signalling proteins within the growth cone or local synthesis of key growth cone components could potentially evoke spatially localized outputs, and thus directed growth and guidance, even in the case of a uniform cue and uniformly localized receptor.

We have shown that growth and guidance occur by a mechanism that favours fluctuations that tend in a direction appropriate to the eventual trajectory of an axon [121,122], but an obvious and fundamental question is what is the source of that spatial bias. This is a subject of active investigation, but a few possible hypotheses can readily be imagined (figure 7). Conceptually, the simplest option is a gradient of a guidance cue. If an external cue is presented in a graded way and thus produces differential levels of signalling activity across the growth cone, this presumably would produce a graded intracellular response, in this case, an internal spatial gradient in actin network connectivity and thus propensity for expansion versus shrinkage of the actin mass with the required polarity. Other mechanisms could produce the same outcome, however. For example, there is compelling evidence that some guidance receptors are localized selectively to the leading versus trailing end of the growth cone, and that this segregation is essential to produce attraction versus repulsion [123]. Differential localization of the responding receptor could produce a spatially graded response within the growth cone even to a uniformly distributed cue, and inverting growth cone polarity—i.e. re-localizing the receptor—would then be predicted to transform a repellant into an attractant and vice versa [124]. A third option that has been observed in other systems is that cue and receptor could both be uniform, but there can be, for example, spatial inhomogeneity in the activity of the signal transduction machinery within a cell or growth cone [125,126], local synthesis of key components of the guidance machinery [127], or localized differences in the mechanical coupling of signalling to the cytoskeleton and substratum. The key to growth cone advance or turning is that there be a spatial bias in the relationship between actin network connectivity and contractility that favours net translocation of the actin in the required direction. Any or all of these mechanisms could provide that bias (as could others); which ones apply in any given case can only be determined experimentally.

In this Commentary, we have discussed only the intrinsic dynamics of the actin cytoskeleton but that does not mean that actin and myosin alone determine axon growth and guidance. It is apparent that the adhesive and biophysical properties of the substratum influence axon growth in some contexts [102,128], and in any given system, their role must be investigated alongside that of actin dynamics. Most important, we have not yet incorporated into the analysis of TSM1 the contribution of the microtubule cytoskeleton, which plays an essential role cooperating with actin in an advancing growth cone [11,35,129]. Various proposals have been offered to account for the role of microtubules in growth cones and the nature of their interactions with actin. It may be, for example, that microtubule extension is mechanically essential to drive growth cone advance, either by pushing forward the leading membrane of the growth cone itself or by providing a pushing force to the actin peak [14,130,131]. Alternatively, or additionally, it may be that a key function of microtubules is simply to prevent the membrane or the actin from regressing once they have extended or to provide a track for delivery of new growth cone components to the leading edge of the axon, such as transmembrane proteins, bulk membrane and actin filaments. To date, these ideas have been studied extensively in the adherent, two-dimensional environment, but the necessary high resolution, dynamic studies have yet to be performed extensively *in vivo*. These are critical subjects for future investigation.

The principles of actin organization and dynamics that we have discussed in this Commentary should apply not only to growth cones and their motility, but generally to cellular structures that are built on actin networks, or are moved by them. There are numerous systems in cell biology in which an actin network grows or shrinks in response to biochemical signals, or in which there must be regulation of the range of action of a mechanical force or the range of transmission of a signal. Many of these could, in principle, be modulated by simple variations on the concepts presented here, and it will be interesting to investigate the range of applicability of these ideas [64,101,112,132,133]. From the perspective of neural development, however, we suggest that the probabilistic view of actin network dynamics we present here offers a new and powerful lens for investigating the properties and mechanisms of axon growth and guidance *in vivo*.

## Data Availability

This article has no additional data.
